# Factors associated with unwillingness to seek professional help for depression: a web-based survey

**DOI:** 10.1186/s13104-017-3010-1

**Published:** 2017-12-04

**Authors:** Eisho Yoshikawa, Toshiatsu Taniguchi, Nanako Nakamura-Taira, Shin Ishiguro, Hiromichi Matsumura

**Affiliations:** 1 Department of Neuropsychiatry, Nippon Medical School Tama Nagayama Hospital, 1-7-1 Nagayama, Tama City, Tokyo 206-8512 Japan; 2 Department of Neuropsychiatry, Nippon Medical School, 1-1-5 Sendagi, Bunkyo, Tokyo 113-8602 Japan; 3 Department of Psychology, Fukuyama University, Sanzo, Gakuen-cho, Fukuyama, Hiroshima 729-0290 Japan; 4 Tottori Seikyo Hospital, 458 Suehiroonsen-cho, Tottori, Tottori 680-0841 Japan; 5 Center for Research on Human Development and Clinical Psychology, Hyogo University of Teacher Education, 2-579-15 Shimokume, Kato-shi, Hyogo, 673-1494 Japan; 6 Ujiie Hospital, 4095 Mukogawara, Sakura-shi, Tochigi, 329-1326 Japan; 7 Specified Nonprofit Organization Depression Support Network, 3-20-11 Tamagawa, Setagaya-ku, Tokyo, 158-0094 Japan

**Keywords:** Barriers to mental health care, Depressive disorder, Web study, Willingness to seek professional help

## Abstract

**Objective:**

Depression is a prevalent disorder that has a substantial impact on not only individuals but also society as a whole. Despite many effective depression interventions, delay in initial treatment contact is problematic. The Internet is a possible tool for low-cost dissemination of appropriate information and awareness raising about depressive disorders among the general public. This study aimed to identify factors associated with unwillingness to seek professional help for depression in Internet users.

**Results:**

This web-based cross-sectional study surveyed 595 participants who scored over the cutoff point for depression on a self-rated mental-health questionnaire for depression, had never been assessed or treated by a mental health professional, and were experiencing depressive symptoms for at least 6 months. Among the 595 participants, 329 (55.3%) reported they were unwilling to seek professional help for depression. Regression analysis indicated that unwillingness to seek professional help for depression was associated with male sex and financial issues as a depression trigger, and that willingness to seek professional help was associated with problems with interpersonal relationships. The Internet warrants further complementary investigation to elucidate factors associated with unwillingness to seek professional help for depression.

## Introduction

Depression is a prevalent disorder that has a substantial impact on not only individuals but also society as a whole [1]. Despite many effective depression interventions, delay in initial treatment contact is problematic [2, 3]. In fact, a longer duration (> 6 months) of untreated depressive symptoms are associated with worse outcomes in depression [3, 4]. Whereas the first step toward appropriate treatment is seeking professional help [5], previous studies have revealed that the most common barrier in those who perceive the need for treatment is a desire to handle the problem on one’s own [6]. When depression seems to be an appropriate reaction to its trigger, rather than perceiving a need for mental health care, they might believe that they should handle the problem’s trigger on their own [7–9]. Therefore, the trigger of depression may play an important role in seeking professional help for depression.

Given that participants with greater attitudinal barriers might have been less willing to participate in this study investigating unwillingness to seek professional help for depression, this research area might underestimate the barriers [10–13]. The Internet is a possible tool for low-cost dissemination of appropriate information and for various intervention programs [14]. Therefore, a web-based survey was needed to provide useful information on depressive disorder through the Internet. Additionally, websites that maintain anonymity might be preferred by people who deal with mental illness alone by choice or otherwise [15]. As a result, a web-based survey may increase generalizability by increasing access to those reluctant to participate in face-to-face surveys [16, 17].

The aim of this study was to elucidate the association between triggers of depression and unwillingness to seek professional help for depression among Internet user experiencing depressive symptoms for at least 6 months and were Internet users with no history of treatment.

## Main text

Participants were informed about the anonymous Internet survey concerning depression by advertisements on the websites of the Depression Support Network, which is a specified nonprofit organization certified by the Tokyo Metropolitan Government (now organization activities thorough web site have shut down and not available in accessing web site). Recruitment took place between December 2010 and February 2011, when participants could access the survey by hyperlink. Inclusion criteria were (1) at least 18 years of age, (2) at least 6-month duration of depressive symptoms, (3) scores higher than the cutoff value of the Depression and Suicide Screen (DSS) [18], (4) never having received treatment for depression, and (5) ability to read and understand Japanese. Exclusion criteria were (1) missing data and/or measurement errors, (2) inappropriate submission (e.g., multiple submission, inconsistent responses), and (3) past history of treatment for depression.

We gathered information on age, sex, vocation, and past history of use of mental health services, all of which were embedded in the survey. We administered DSS to assess depression. DSS is a self-report questionnaire consisting of 5 items. The reliability and validity of the DSS have been verified, and a cutoff of 1/2 was reported to be suitable [18]. Duration of depression was assessed using the embedded question “How long have you been feeling depressed?” We used embedded non-validated questionnaires to assess willingness to seek professional help and depression triggers. The items used to assess willingness to seek professional help and triggers of depression were selected through consultation with psychiatrists, other medical doctors, and the staff of Depression Support Network. Some members of the Depression Support Network staff had experienced depressive episodes and received psychiatric treatment. Regarding treatment of depression, clinical psychologists in Japan are not yet certified or nationally accredited to provide such services. Therefore, it is not always easy to seek help from clinical psychologists directly. Further, there are few doctors who specialize in primary care because the medical system has no clear definition of primary care or the specific providers responsible for it [19, 20]. Therefore, treatment of depression is usually provided by psychiatrists rather than general practitioners. Willingness to seek professional help was measured by two questionnaire items. The first asked, “People differ a lot in their feelings about professional help for emotional problems. Would you seek professional help, for example, from a psychiatric doctor or family doctor?” Responses are a dichotomous variable: (a) not willing to seek help, (b) willing to seek help from a psychiatric (or psychosomatic) doctor, and (c) family doctor. Next, they were asked, “Do you want to receive psychotherapy from a clinical psychologist?” Responses are a dichotomous variable: (a) do not want to receive psychotherapy, and (b) want to receive psychotherapy. The questions about triggers of depression were embedded in the questionnaire as follows: “What are the cause and triggers of your depression?” (1) problem in the workplace (yes/no); (2) family problem (yes/no); (3) problem in interpersonal relationships (yes/no); (4) financial problem (yes/no); (5) physical illness (yes/no); (6) separation from family member (yes/no); (7) none (yes/no); (8) others (yes/no) (multiple responses permitted).

### Statistical analysis

We tested the difference between unwillingness and willingness to seek professional help using the χ^2^ test and *t* test as appropriate to the variable. Next, we conducted multiple binary logistic regression analysis with the backward elimination method to explore factors associated with unwillingness to seek professional help. In the backward elimination method, dependent variables were unwillingness and willingness to seek professional help and independent variables were sex, age, vocation type, duration of depressive symptoms, age at onset, and depression triggers. All analyses were performed using SPSS, version 23 (SPSS Inc., Chicago). Alpha level for two-tailed tests was set at *p* < 0.05.

## Results

We obtained informed consent from 4455 respondents through the website. We excluded questionnaires of 2817 respondents for inappropriate submission, missing data, and/or measurement errors. Of the remaining 1638 respondents, 825 had a score for depression above the cutoff point. Of these, 617 had experienced depressive symptoms for over 6 months. We then excluded 22 participants with a history of psychiatric treatment for depression. As a result, data of 595 participants were included in the statistical analysis.

Table 1 shows participants’ variables, demographic information, and results of binary analysis. Unwillingness to seek professional help for depression was significantly associated with sex and financial issues as a depression trigger. Among the 595 participants, 329 (55.3%) reported their unwillingness to seek professional help for depression and 266 (44.7%) reported their willingness to seek professional help for depression in the past (from psychiatrists, *n* = 97 [36.5% of all the participants, 16.3% of participants with willingness to seek professional help]; family doctor, *n* = 18 [6.5, 3%]; clinical psychologists, *n* = 246 [41.3, 92.5%]). Table 2 shows the results of multiple regression analysis. Backward selection binary logistic regression analysis indicated that unwillingness to seek professional help for depression was significantly associated with male sex, older age, and the depression trigger of financial issues, and that willingness to seek professional help was significantly associated with problems with interpersonal relationships.

**Table 1:** Demographic characteristics of participants (*n* = 595) and results of binary analysis

	Total		Willingness to seek professional help for their depression				t	p
	N = 595		No (n = 329)		Yes (n = 266)			
	Mean	SD	Mean	SD	Mean	SD		
Age	34.9	10.2	35.4	10.1	34.5	10.2	1.09	0.28
Age at onset	30.9	10.4	30.4	10.4	31.5	10.3	1.29	0.20
Duration of depressive symptoms without seeking professional help (months)	48.0	36.5	49.0	39.3	46.7	38.1	− 0.72	0.47

	Total		Willingness to seek professional help for their depression				χ	p
			No		Yes			
	N	%	n	%	n	%		
Sex								
Male	268	45.0	170.0	63.4	98.0	36.6	13.07	< 0.01*
Female	327	55.0	159.0	48.6	168.0	51.4		
Vocation								
Student								
Yes	52	8.7	30.0	57.7	22.0	42.3	0.13	0.72
Company worker								
Yes	269	45.2	146.0	54.3	123.0	45.7	0.21	0.65
Public officer								
Yes	11	1.8	7.0	63.6	4.0	36.4	0.32	0.57
Independent business								
Yes	35	5.9	19.0	54.3	16.0	45.7	0.02	0.90
Homemaker								
Yes	117	19.7	54.0	46.2	63.0	53.8	4.92	0.03
None								
Yes	41	6.9	26.0	63.4	15.0	36.6	0.00	0.96
Trigger of depression								
Work problem								
Yes	350	58.8	196.0	56.0	154.0	44.0	0.17	0.68
Family issue								
Yes	216	36.3	116.0	53.7	100.0	46.3	0.35	0.56
Any interpersonal relationship								
Yes	173	29.1	88.0	50.9	85.0	49.1	1.93	0.16
Financial difficulties								
Yes	311	52.3	186.0	59.8	125.0	40.2	5.37	0.021*
Physical illness								
Yes	128	21.5	63.0	49.2	65.0	50.8	2.44	0.12
Separation from family member								
Yes	14	2.4	9.0	64.3	5.0	35.7	0.47	0.49
None								
Yes	41	6.9	26.0	63.4	15.0	36.6	1.18	0.28

* *p* < 0.05

**Table 2:** Results of multiple logistic regression analysis to assess factors associated with unwillingness to seek professional help

	B	SE	Wald	p	OR	95% CI
Sex	− 0.638	0.176	13.188	0.001*	0.528	0.374–0.745
Age	− 0.021	0.009	5.808	0.016*	0.979	0.963–0.996
Trigger of depression						
Financial difficulties	0.403	0.174	5.348	0.021*	1.496	1.063–2.104
Interpersonal relationships	− 0.396	0.191	4.305	0.038*	0.673	0.463–0.978

*SE* standard error, *OR* odds ratio, *95% CI* 95% confidence interval

* *p* < 0.05

## Discussion

In current survey, approximately 55.3% of the Internet users in the current study were unwilling to seek professional help. And the findings suggest that unwillingness to seek professional help for depression was associated with depression triggers in Internet users. Financial difficulties might hinder them from seeking professional help. In fact, financial difficulty is reported to be associated with suicide, and it is the second leading cause of suicide in Japan [21]. A review of coroners’ records indicated that individuals whose deaths were thought to be related to an economic recession were less likely to have a history of self-harm but were more likely to have financial difficulties, and less than 20% of these individuals had had any contact with psychiatric service providers [22]. In contrast, interpersonal problems might encourage individuals to seek professional help. We found that over 40% of all participants preferred to seek help from a clinical psychologist. Some evidence suggests that psychotherapy and counseling are preferable to antidepressants in the treatment of depressive disorder, especially in Japan [23, 24]. The findings might suggest a potential need for psychotherapy in Internet users who explore websites for coping with depression.

It was also suggested that male sex and age were associated with an unwillingness to seek professional help for depression. A Japanese survey previously reported that female sex was significantly associated with an unwillingness to seek professional help [25]. However, a cross-national survey demonstrated that females and younger people with disorders were more likely to recognize a need for treatment, and that major barriers to seeking treatment were a low perceived need and attitudinal barriers [6]. Discrepancies between findings might reflect the characteristics of Internet users in Japan. The inconsistent findings of previous Japanese surveys might imply the need for a different strategy for providing information on depression via the Internet. The findings of the current study might suggest that both financial difficulty as a depression trigger and the reader’s need for psychotherapy should be taken into account when disseminating information on depression on the Internet. A strength of this survey is the substantial number of participants, who cannot be easily reached by mental health researchers or providers. Ease of access to a large number of participants is one of the advantages of a web survey [16]. In the current study, participants’ actual need for psychiatric treatment was unknown. Mean duration of depressive symptoms was more than 4 years, which suggests the potential need for appropriate assessment and treatment by a mental health provider. It might be suggested that a web-based survey was useful for investigating the associated factor of unwillingness to seek professional help for depression.

## Limitations

This study has several limitations. Our findings do not generalize to those without computers or access to a computer network. Furthermore, selection bias and limits of external validity are unknown. For example, socially disadvantaged groups might be underrepresented among Internet users, as income was associated with network milieu, such as ownership of a personal computer and/or the availability of Internet access. Higher income is also associated with higher levels of education, which leads to early adoption of information technology [14]. Another factor to be taken into account is age. Elderly people, who often have difficulty using new technology, might be underrepresented among Internet users [26]. Reduced or absent interaction with participants during a web survey creates problems if instructions are misunderstood. Second, Sampling might be dependent on public awareness of the Depression Support Network and exposure to other resources, such as educational seminars on depression or telephone counseling. Sampling may have also been influenced by the order in which search engines returned relevant results to users’ queries (i.e., making them more or less likely to encounter the website). Third, our findings might have been biased by the non-validated questionnaires used to assess willingness to seek professional help, triggers of depression, preference for using services other than visiting a mental health provider. For example, possible triggers of depression did not include some important variables such as “death of a loved one” Furthermore, recall bias is inevitable given the nature of retrospective self-report surveys. Fourth, a cross-sectional study design cannot establish causality between factors. Fifth, this study did not systematically assess important variables related to unwillingness to go to a mental hospital, such as education, marital status, and perceived stigma. Finally, residual confounding by uncontrolled or unmeasured factors might have distorted genuine associations. In conclusion, the Internet warrants further complementary investigation to elucidate factors associated with unwillingness to seek professional help for depression.

## Abbreviations

DSS: Depression and Suicide Screen
SD: standard deviation
SE: standard error
OR: odds ratio
95% CI: 95% confidence interval
